# Adjuvants and the vaccine response to the DS-Cav1-stabilized fusion glycoprotein of respiratory syncytial virus

**DOI:** 10.1371/journal.pone.0186854

**Published:** 2017-10-26

**Authors:** Mallika Sastry, Baoshan Zhang, Man Chen, M. Gordon Joyce, Wing-Pui Kong, Gwo-Yu Chuang, Kiyoon Ko, Azad Kumar, Chiara Silacci, Michelle Thom, Andres M. Salazar, Davide Corti, Antonio Lanzavecchia, Geraldine Taylor, John R. Mascola, Barney S. Graham, Peter D. Kwong

**Affiliations:** 1 Vaccine Research Center, National Institute of Allergy and Infectious Diseases, National Institutes of Health, Bethesda, Maryland, United States of America; 2 Institute for Research in Biomedicine, Bellinzona, Switzerland; 3 The Pirbright Institute, Ash Road, Pirbright, Woking, Surrey GU24 0NF, United Kingdom; 4 Oncovir Inc., Washington, D.C, United States of America; 5 Humabs BioMed SA, Bellinzona, Switzerland; University of Iowa, UNITED STATES

## Abstract

Appropriate adjuvant selection may be essential to optimize the potency and to tailor the immune response of subunit vaccines. To induce protective responses against respiratory syncytial virus (RSV)—a highly prevalent childhood pathogen without a licensed vaccine—we previously engineered a pre-fusion-stabilized trimeric RSV F (pre-F) “DS-Cav1” immunogen, which induced high titer RSV-neutralizing antibodies, in mice and non-human primates, when formulated with adjuvants Poly (I:C) and Poly (IC:LC), respectively. To assess the impact of different adjuvants, here we formulated RSV F DS-Cav1 with multiple adjuvants and assessed immune responses. Very high RSV-neutralizing antibody responses (19,006 EC_50_) were observed in naïve mice immunized with 2 doses of DS-Cav1 adjuvanted with Sigma adjuvant system (SAS), an oil-in-water adjuvant, plus Carbopol; high responses (3658–7108) were observed with DS-Cav1 adjuvanted with Alum, SAS alone, Adjuplex, Poly (I:C) and Poly (IC:LC); and moderate responses (1251–2129) were observed with DS-Cav1 adjuvanted with the TLR4 agonist MPLA, Alum plus MPLA or AddaVax. In contrast, DS-Cav1 without adjuvant induced low-level responses (6). A balanced IgG1 and IgG2a (Th2/Th1) immune response was elicited in most of the high to very high response groups (all but Alum and Adjuplex). We also tested the immune response induced by DS-Cav1 in elderly mice with pre-existing DS-Cav1 immunity; we observed that DS-Cav1 adjuvanted with SAS plus Carbopol boosted the response 2-3-fold, whereas DS-Cav1 adjuvanted with alum boosted the response 5-fold. Finally, we tested whether a mixture of ISA 71 VG and Carbopol would enhanced the antibody response in DS-Cav1 immunized calves. While pre-F-stabilized bovine RSV F induced very high titers in mice when adjuvanted with SAS plus Carbopol, the addition of Carbopol to ISA 71 VG did not enhance immune responses in calves. The vaccine response to pre-F-stabilized RSV F is augmented by adjuvant, but the degree of adjuvant-induced enhancement appears to be both context-dependent and species-specific.

## Introduction

Human respiratory syncytial virus (RSV) infection is the most common cause of hospitalization for lower respiratory tract infection (LTRI) in children under five years of age, worldwide. Severe RSV disease occurs at the extremes of age. RSV is the leading cause of death due to LTRI in children under six months of age [1]. Among elderly patients, RSV infection is also a major cause of hospitalizations and associated deaths in the USA [2–4]. Thus, the development of an effective RSV vaccine is of substantial importance. Live vaccines such as the smallpox vaccine pioneered by Jenner [5] provide immunity, but may have safety risks; inactivated vaccines are generally safer, but when a formalin-inactivated RSV vaccine adjuvanted with alum was evaluated in healthy infants and young children in the 1960’s [6, 7] 80% of vaccinees who were infected required hospitalization compared to 5% of the control group.

Considerable effort has been directed towards developing subunit -based RSV vaccines designed to elicit potently neutralizing antibodies targeting specific epitopes. RSV F and G surface proteins as well as chimeric F/G variants when used as immunogens, resulted in robust albeit poorly neutralizing antibodies [8, 9]. These subunit vaccine approaches have been further explored in both purified protein [10–13] and vector-based formats [14, 15]. Vaccine approaches based on soluble proteins are generally poorly immunogenic and usually require adjuvants to augment their immunogenicity. A number of synthetic and natural compounds have been identified to have adjuvant activity, however, only a few including alum, squalene oil-in-water (MF59), and monophosphoryl lipid A (MPLA) have achieved widespread human use. Most adjuvants either activate pattern recognition receptors (PRRs, such as toll-like receptors (TLRs)) in the innate immune system or improve the delivery of antigens to the immune system. The most common adjuvant, alum, comprised of aluminum salts, has been used in humans since 1932, is approved for human use by the FDA, and is a component of numerous licensed vaccines such as Diphtheria, Tetanus and Pertussis (DTaP) vaccines, and hepatitis B vaccines. MPLA with Alum is used for the hepatitis B vaccine, Fendrix, and the human papillomavirus (HPV) vaccine, Cervarix, and has extensive human safety data in this context. Oil-in-water formulations such as MF59 [16] are components of FLUAD (a new seasonal flu vaccine for the elderly) MPLA is also a component of Pollinex Quattro, a vaccine used for the treatment of seasonal allergic rhinitis [17]. Combinations of MF59 with polyanionic carbomers [16, 18] as well as Adjuplex (a coformulation of carbomer with lecithin [19]) are potent and well-tolerated adjuvants when administered with subunit vaccines. Carbomers have also been evaluated as experimental adjuvants in veterinary vaccines against swine parvovirus [20], circovirus type 2 [21], *Staphylococcus aureus* in sheep [22], and equine influenza virus [23]. Poly (I:C), a synthetic double-stranded RNA and its derivative Poly (IC:LC) [10, 24] have been reported in a range of animal species from mice to nonhuman primates revealing significant enhancement of immunogenicity and are in cancer immunotherapy clinical trials [25] and are a component of intranasal flu vaccine FluMist [26]. Montanide is a family of oil-based adjuvants used to produce an emulsion with the antigen of interest. It has been used in experimental vaccines in mice, rats, dogs and cats using natural, recombinant and synthetic antigens. Montanide is used worldwide in both bovine and ovine vaccinations against viral, bacterial or parasitic diseases. In humans, Montanide emulsions have been used in experimental human vaccines against HIV, malaria and breast cancer [27, 28]. Each of these adjuvants have unique characteristics and mechanisms, and their impact on the immune response induced by a particular immunogen generally needs to be determined experimentally.

We previously engineered a pre-fusion stabilized trimeric RSV F (pre-F) immunogen, named “DS-Cav1”. DS-Cav1 when adjuvanted with TLR3 agonists Poly (I:C) and Poly (IC:LC) induces high neutralizing antibody titers in mice and nonhuman primates, respectively [10]. Herein, we report a comparative adjuvant study with DS-Cav1 formulated with nine different adjuvants (Table 1) in naïve mice. We also studied elderly mice that had been pre-immunized with DS-Cav1 adjuvanted with Poly (I:C). We further evaluated the impact of Carbopol in mice and calves using a bovine RSV F version of DS-Cav1. The results indicate that adjuvant is required for DS-Cav1 to induce high titer responses, that moderate to high titer responses can be induced by all the adjuvants tested, and that the optimal response to adjuvants appears to be both context-dependent (e.g. whether animals have pre-existing responses) and species-specific (e.g. the addition of Carbopol induced very high responses in mice, but not in calves).

**Table 1:** Adjuvants and their proposed mode of action.

Adjuvant	Characteristics	Putative mechanism, receptor, type of immune response
Aluminium salts (Aluminium hydroxide)	Gel suspension	Inflammasome activation; Th2 biased response [29],[30]
Poly (I:C)	dsRNA (Inosine, cytidine homologue)	TLR3 Agonist (immunomodulatory molecule) [31]
Poly (IC:LC)	dsRNA (Complex of carboxymethylcellulose, polyinosinic-polycytidylic, and poly-L-lysine double-stranded RNA)	TLR3 Agonist (immunomodulatory molecule) [24]
Monophosphoryl Lipid A-SM	Phospholipid	Surface PRR activation; TLR4 agonist, Immunomodulatory molecule [32] [33, 34].
Sigma Adjuvant System (SAS)	Oil-in-water emulsion, MPLA and trehalose dicorynomycolate	Enhanced antigen uptake; TLR4 agonist
MPLA + Alum	Phospholipid plus gel suspension	TLR4 agonist, combination
SAS + Carbopol	Oil-in-water emulsion, MPLA, trehalose dicorynomycolate. Polyanionic high mol. weight acrylic acid	TLR4 agonist and controlled release of antigen[21]
Adjuplex	Lecithin & Carbomer (cross-linked polyacrylic acid) homopolymer	Th1/Th2 balanced response [19]
AddaVax	Squalene based Oil in Water Emulsion	Th1/Th2 balanced response[35]
Montanide^TM^ ISA 71 VG	Water in oil emulsion	Th1/Th2 balanced response
Montanide^TM^ ISA 71 VG + Carbopol	Water in oil emulsion. Polyanionic high mol. weight acrylic acid	Th1/Th2 balanced response and controlled release of antigen

## Materials and methods

### Adjuvants

Alhydrogel (Alum) was purchased from Brenntag, Denmark, AddaVax, Monophosphoryl Lipid A-SM (MPLA) and Poly (I:C) were purchased from Invivogen, Sigma Adjuvant System (SAS) and Adjuplex were purchased from Sigma-Aldrich, Carbopol was a gift from Mutchler Inc, Poly (IC:LC) was a gift from A.M. Salazar, Oncovir, DC. Montanide ISA 71 VG was a gift from Seppic, France.

### Cell lines, resins and proteases

Expi293F cells and 293fectin were purchased from Thermo Fisher scientific, Ni Sepharose Excel and Strep-Tactin Superflow resins were purchased from GE Healthcare and IBA, respectively. Restriction grade thrombin was obtained from Novagen. HEp2 cells were purchased from ATCC, VA.

### Protein expression and purification

RSV F DS-Cav1 [10] and RSV F DS-Cav1 site Ø (K65N-N67T, P205N-V207T, K209N-S211T) and site II (N268R-K272E) knock-out (KO) proteins were expressed by transient transfection of Expi293F cells using 293fectin. The culture supernatant was harvested 5 days post transfection and centrifuged at 10,000 *g* to remove cell debris. The supernatant was sterile-filtered and RSV F DS-Cav1 was purified by Nickel and Strep-Tactin-affinity chromatography followed by size-exclusion chromatography using a Superdex 200 16/60 column (GE Healthcare). The His_8_ and Strep-Tactin purification tags were removed from the RSV F protein by digestion with restriction-grade biotinylated thrombin (Novagen) overnight at 4°C. Cleaved RSV F DS-Cav1 was further purified by size-exclusion chromatography (SEC) in phosphate-buffered saline (PBS) prior to immunization. Bovine RSV F DS-Cav1 [36] was expressed and purified analogously. Specifically, bovine RSV F DS-Cav1 was expressed by transient transfection of Expi293F cells using 293Fectin and purified using IMAC, Strep-Tactin affinity and size-exclusion chromatography.

### Ethics statement

All mouse experiments were reviewed and approved by the Animal Care and Use Committee of the Vaccine Research Center, NIAID, NIH, under animal protocol identification number 13–454, and all animals were housed and cared for in accordance with local, state, federal, and institute policies in an American Association for Accreditation of Laboratory Animal Care (AAALAC)-accredited facility at the NIH. The calf experiment was performed under the regulations of the United Kingdom Home Office Scientific Procedures Act (1986). The study was reviewed and approved by the Animal and Plant Health Agency (APHA) Ethical Review Committee.

### Mouse immunizations

CB6F1/J female hybrid mice used in our study are the first filial offspring of a cross between BALB/cJ females (C) and C57BL/6J males (B6) (The Jackson Laboratory). These mice were used in our earlier RSV immunogenicity experiments; provide increased genetic complexity and flexibility and allow direct comparison to our prior results. Mice at age 10 weeks were injected with RSV F DS-Cav1-adjuvant mixtures at week zero and week three for homologous prime-boosts into the semimembranosus muscle of both pelvic limbs with 10 μg of RSV F DS-Cav1 immunogen with or without adjuvants. All immunizations were given intramuscularly; each animal received in total 100μl of PBS containing 10 μg of RSV F DS-Cav1 immunogen: in 100μl inoculant with 50 μl (50% v/v) mixture of Sigma Adjuvant System (SAS) (Sigma) for the SAS group; in 100μl inoculant with 50 μg (5 fold w/w) of Poly (I:C) for the Poly (I:C) group; in 100μl inoculant with 50 μg (5 fold w/w) of Poly (IC:LC) for the Poly (IC:LC) group; in 100μl inoculant with 20 μg (2 fold w/w) of Monophosphoryl Lipid A for the MPLA group; in 100μl inoculant with 100 μg (10 fold w/w) of Alum for the Alum group; in 100μl inoculant with 50 μl (50% v/v) mixture of Sigma Adjuvant System plus 20 ng (0.02% w/v) of Carbopol for the SAS + Carbopol group; in 100μl inoculant with 50 μl (50% v/v) of AddaVax for the AddaVax group; in 100μl inoculant with 20 μl (20% v/v) of Adjuplex for the Adjuplex group; in 100μl inoculant with 20 μg (2 fold w/w) of Monophosphoryl Lipid A plus 100 μg (10 fold w/w) of Alum for the MPLA + Alum group. Inoculants were formulated within one hour before immunization followed by mixing immediately prior to injections. A control group with no adjuvant was also included in the study. No adverse effect from immunization was observed in any study animal. Sera samples were collected at least three days before immunization, and two weeks after each immunization or any indicated time points. Animal experiments were conducted in full compliance with all relevant federal regulations and NIH guidelines. For the elderly mice study, two groups of seven mice that had been pre-immunized multiple times with DS-Cav1: Poly (I:C) formulation were immunized twice with DS-Cav1 formulated with Alum and SAS + Carbopol after a waiting period of ~83 weeks. As with naïve mice, two injection sites in the semimembranosus muscle of both pelvic limbs were used for each immunization. No adverse effect from immunization was observed. Sera were collected at least three days before immunization, and at weeks two and weeks five post initial immunization.

### Calf immunizations

BRSV seronegative male calves were produced as described previously [36] and vaccinated intramuscularly with 50 μg of bRSV F DS-Cav1 adjuvant mixtures in a volume of 2 ml on two occasions 4 weeks apart. Calves were 3 to 6 weeks of age at the time of vaccination. Five calves were vaccinated with bRSV F DS-Cav1 emulsified with Montanide^TM^ ISA 71 VG (Seppic, France) in a water in oil emulsion at a ratio of 70:30 adjuvant to aqueous phase, as described previously [36] and two calves were vaccinated with bRSV F DS-Cav1 plus Carbopol 1:1 v/v emulsified with Montanide^TM^ ISA 71 VG 1:1 v/v. Calves developed either mild or no swelling at the injection sites and a transient fever 24 h after vaccination. Sera were collected at one to two weekly intervals after vaccination.

### Viruses and cells for RSV neutralization assays

Viral stocks were prepared and maintained as previously described [37]. Recombinant mKate-RSV expressing prototypic subtype A (strain A2) F genes and the Katushka fluorescent protein were constructed as reported by Hotard et al. [38]. HEp-2 cells (ATCC, VA) were maintained in Eagle's minimal essential medium containing 10% fetal bovine serum (10% EMEM), supplemented with glutamine, penicillin and streptomycin.

### RSV neutralization assays

The neutralization antibody was measured by a fluorescence plate reader neutralization assay. 2.4 x 10^4^ HEp-2 cells/well in 30 μl culture medium were seeded in 384-well black optical bottom plate (Nunc®384-well plates, Thermo Scientific, West Palm Beach, FL). When palivizumab (Synagis®) is dosed at a concentration of 15 mg/kg, serum levels at trough are ~40 μg/ml, which provides protection in infants from severe disease and protection in cotton rats from RSV infection. In our neutralization assay, ~40 μg/ml of palivizumab yields an EC_50_ of 100 [10]. Serum samples were diluted in four-fold dilutions starting from 1:10 to 1: 163840, an equal volume of recombinant mKate-RSV A2 was added and mixed, incubated at 37°C for one hour, after incubation, 50 μl mixture of sample and virus was added to cells/well in the 384-well plate, which was incubated at 37°C for 22–24 hours. After incubation, assay plates were analyzed for fluorescence intensity in a microplate reader at an excitation wavelength of 588 nm and emission wavelength of 635 nm (SpectraMax Paradigm, Molecular Devices, Sunnyvale, CA 94089). EC_50_ of neutralization of sample was calculated by curve fitting using GraphPad Prism 6 software (GraphPad Software Inc., San Diego. CA).

### Bovine RSV neutralization assays

BRSV microneutralization assay was performed using 500–1,000 TCID50 (50% tissue culture infectious doses) of bRSV, strain 375 (ATCC VR1339) and BT cells (ATCC CRL1390) [36]. Briefly, immune sera were serially diluted in quadruplicates prior to mixing with 500–1000 TCID50 of bRSV for 1 h at 37°C in a humidified 5% CO_2_ atmosphere prior to addition to monolayers of BT cells seeded the day before at 8000 cells/well. Cells were then incubated for 7 days, fixed with 70% methanol, stained with 1% crystal violet and examined at the microscope for syncytia formation and cytopathic effect (CPE). Neutralizing titer was defined as the reciprocal of the highest sera dilution at which the infectivity of bRSV was completely neutralized in 50% of the wells. Infectivity was identified by the presence of CPE and syncytia on day 7, and the titer was calculated by the Reed- Muench method [39].

### Sera binding analysis

A fortéBio Octet Red384 instrument was used to measure sera recognition of DS-Cav1, DS-Cav1 antigenic site Ø KO, DS-Cav1 antigenic site II KO, RSV post F and RSV post F site II KO proteins [40] to probe antigenic site Ø (D25, AM22) and site II (motavizumab) targeting antibodies. A series of scouting experiments at different sera dilutions, buffer conditions and sensor tips were performed to minimize non-specific binding. Optimum non-specific binding was obtained using HIS1K sensor tips and sera dilutions in the range of 1:100–1:300 in PBS supplemented with 1% BSA. Initial assays using sera from 10 groups of mice that had been immunized with DS-Cav1: adjuvant formulations as well as the pre-bleed sera with the three pre-fusion specific and two post fusion specific probes were performed at 30°C in tilted black 384-well plates (Geiger Bio-One) with agitation set to 1,000 rpm in PBS supplemented with 1% BSA and a well volume of 55 μl. All experiments were performed in duplicate and included 6–10 mice samples per group based on sera availability. DS-Cav1 and DS-Cav1 KO and Post F KO probes (20 μg/ml) were immobilized for 300s on HIS1K biosensor tips and equilibrated for 60 s in 1% BSA/PBS prior to measuring association with sera diluted 1:100 in BSA/PBS. Variability in loading within a row of eight tips did not exceed 0.1 nm for each of these steps. Parallel correction to subtract systematic baseline drift was carried out by subtracting the measurements recorded for a loaded sensor incubated in PBS + 1% BSA. The association after 300 s was recorded. A similar assay was used to measure sera reactivity for the two groups of pre-immunized mice at ~85 weeks post immunization as well as the two boosts to DS-Cav1, Post F, DS-Cav1 site Ø KO, DS-Cav1 site II KO and Post F site II KO probes. Data analysis was performed using Octet and GraphPad Prism 6 software.

### Measurement of IgG response by ELISA

Maxisorp plates (Nunc, 96 well) were coated with DS-Cav1 at 1μg/ml in PBS at 4°C overnight. Plates were washed and blocked [41] and incubated with serial dilutions of mouse sera from the 10 different groups, pre-bleed sera from each group was included as a negative control. Anti-mouse IgG1 or IgG2a-conjugated with horse radish peroxidase were used as secondary antibodies and 3, 5, 3´5´-tetramethylbenzidine (TMB) was used as the substrate to detect RSV F specific IgG1 or IgG2a antibody responses. Endpoint titers were calculated as the highest serum dilution that gave an optical density exceeding five times the background. ELISA was performed in duplicate and included 6–10 mice samples per group based on sera availability and endpoint titers were averaged and analyzed using GraphPad Prism 6 software. A similar assay performed in duplicate, was used to measure IgG response in the two groups of seven elderly mice at ~85 weeks post immunization as well as the two post boost 1 and post boost 2 time points. Endpoint titers were averaged and analyzed using GraphPad Prism 6 software.

## Results

### Immunogenicity of RSV F DS-Cav1-adjuvant formulations in mice

**Fig 1:**
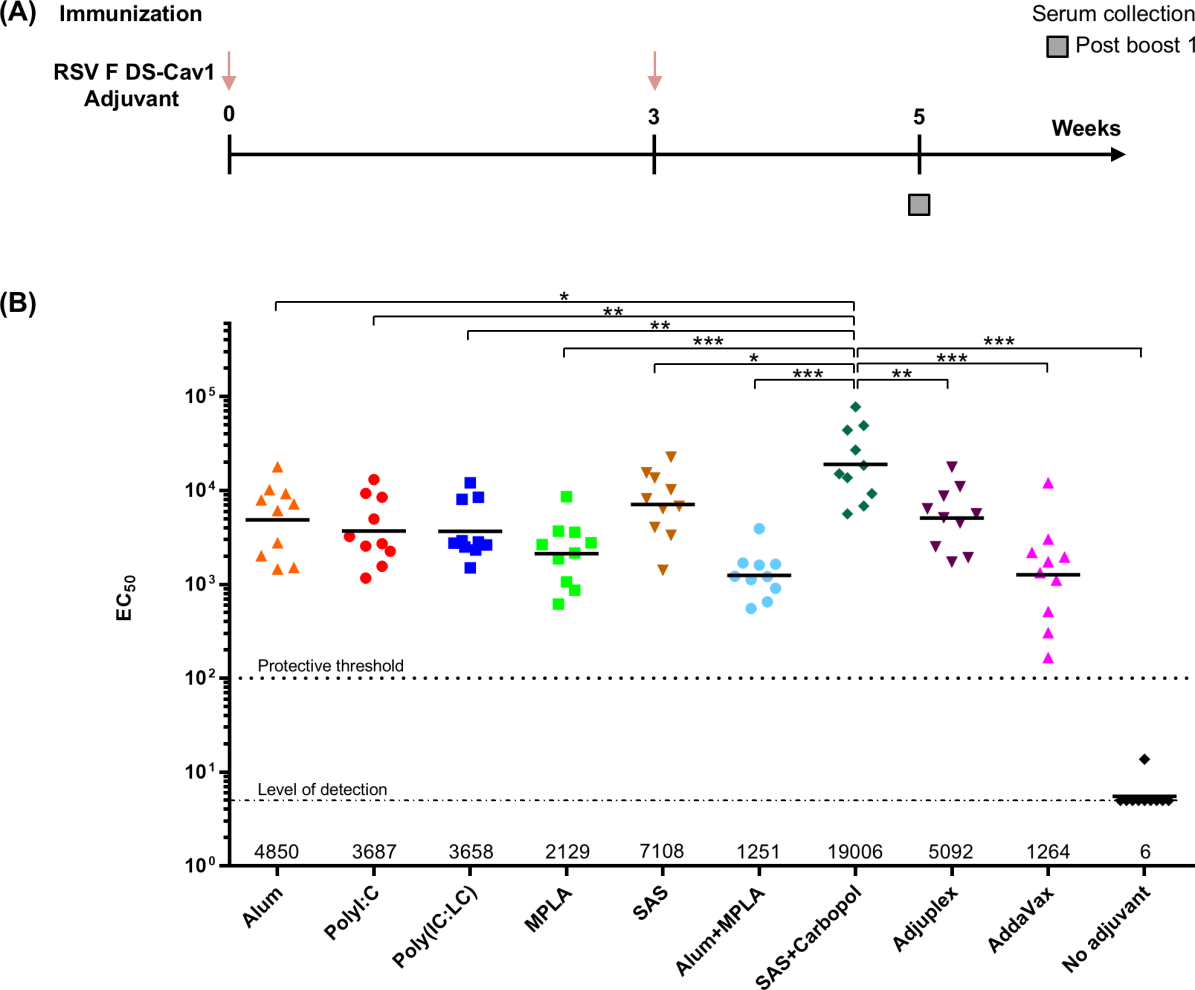
Adjuvants augment DS-Cav1 response in mice. **(A)** RSV F (pre-F) DS-Cav1 formulated with nine different adjuvants was used to immunize mice at an interval of 3 weeks and **(B)** neutralization titers for all nine adjuvanted plus the control unadjuvanted groups are shown. Scatter plots show the geometric mean (numerical value below), each group included 10 mice. The palivizumab protective threshold is indicated by a dotted line [10] and *p* values for SAS + Carbopol/DS-Cav1 versus the other eight adjuvant formulations as assessed by two-tailed Mann-Whitney test and adjusted for multiple comparisons using the Holm-Bonferroni method; *p =* > 0.05 (ns); *p* < 0.05 (*); *p* < 0.01 (**); *p* < 0.001 (***) are shown. All adjuvanted groups show *p* < 0.0001 when compared to the no adjuvant group. Associated raw data is reported in S1 Table.

DS-Cav1-adjuvant mixtures were used to immunize 10 groups of 10 CB6F1/J mice. For each group, 10 μg of DS-Cav1 was formulated with nine different adjuvants and injected intramuscularly twice at an interval of three weeks (Fig 1A). After two immunizations, in the week five sera, significantly higher EC_50_ neutralization titers were observed in mice immunized with DS-Cav1 adjuvanted with Alum, TLR3 agonists Poly (I:C), Poly (IC:LC), TLR4 agonists SAS, SAS + Carbopol, a polyanionic high molecular weight acrylic acid, and Adjuplex, an adjuvant based on purified lecithin and carbomer homopolymer (with geometric mean titers of 4850, 3687, 3658, 7108, 19006, 5092, respectively) than for DS-Cav1 adjuvanted with TLR4 agonist MPLA, Alum plus MPLA and AddaVax (with geometric mean titers of 2129, 1251, and 1264, respectively, Fig 1B). Neutralization titers for the control unadjuvanted DS-Cav1 were below the protective threshold [10] and at the level of detection (Fig 1B). Titers for DS-Cav1 adjuvanted with a combination of SAS, a TLR4 agonist and Carbopol were 15-fold higher than that observed for the lowest neutralizing group represented by DS-Cav1 adjuvanted with Alum plus MPLA (*p* < 0.0009) and four to five-fold higher than DS-Cav1 adjuvanted with Alum (*p* = 0.0136) or the TLR3 agonists Poly (I:C) (*p* = 0.0028), or Poly (IC:LC) (*p* = 0.0025), respectively. Interestingly, the TLR4 agonist MPLA by itself or when formulated with Alum did not help elicit high neutralization titers. Conversely, the TLR4 agonist present as a component in SAS and SAS + Carbopol adjuvants helped elicit the highest immune response among the nine adjuvant groups.

### RSV F DS-Cav1-adjuvant formulations direct the neutralizing response to antigenic sites Ø, II

**Fig 2:**
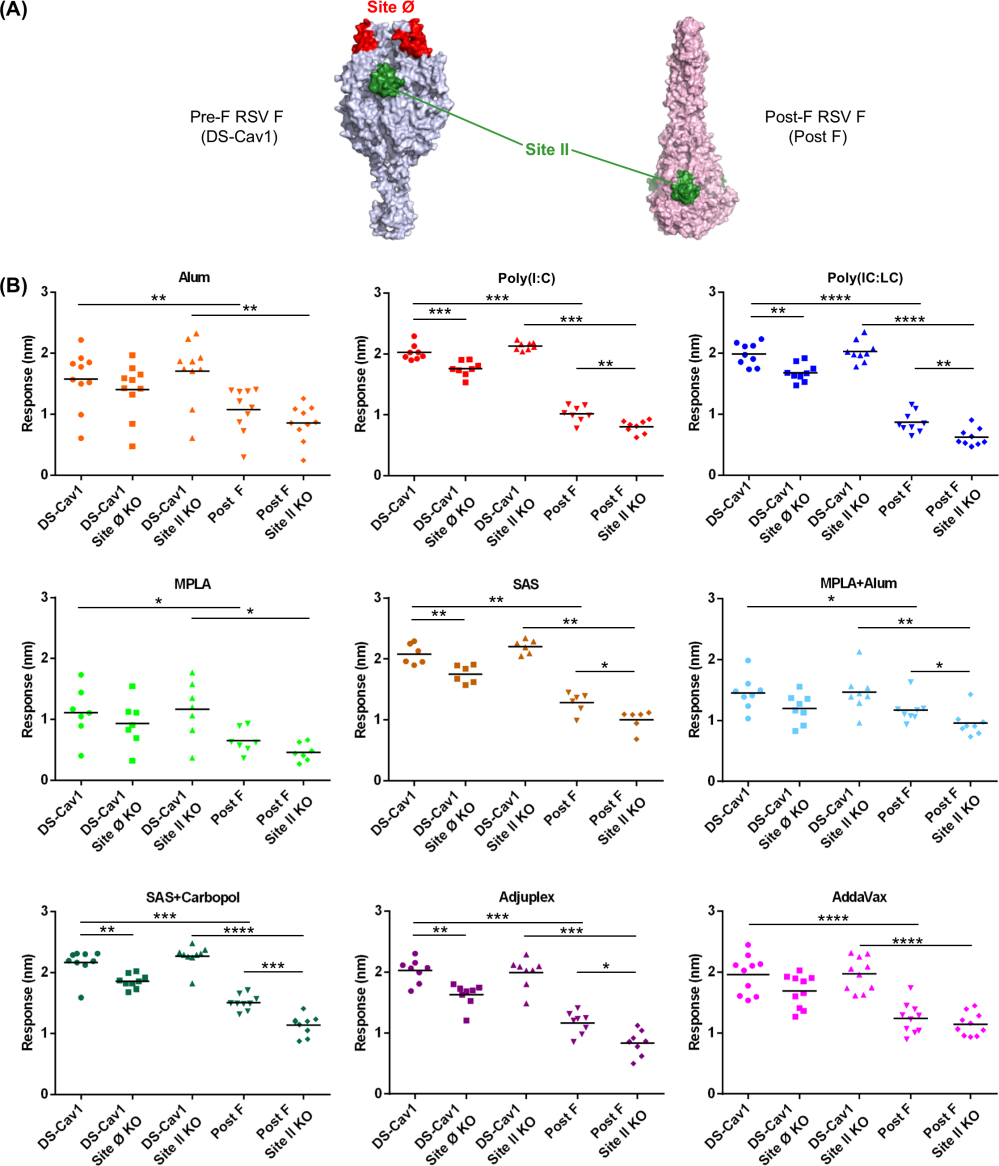
Sera from mice immunized with DS-Cav1-adjuvant mixtures exhibit differential antibody responses to sites Ø and II KO probes. **(A)** Unique and shared antigenic sites on pre-and post RSV F. Antigenic site Ø, a major target of neutralizing antibodies (e.g. D25) and antigenic site II present in both conformations of RSV F are shown on the pre-F and post-F structures. **(B)** Recognition of pre-fusion forms of RSV F (DS-Cav1), DS-Cav1 site Ø KO and site II KO probes by week 5 sera for the nine DS-Cav1 adjuvant groups. Scatter plots show the geometric mean. Pre-bleed sera from 10 mice were used as control. *p* values for DS-Cav1 versus DS-Cav1 site Ø, site II KO probes and Post-F probes as assessed by two-tailed Mann-Whitney test; *p* = > 0.05 (ns); *p* < 0.05 (*); *p* < 0.01 (**); *p* < 0.001 (***) are shown. Associated raw data is reported in S2 Table.

To understand the higher neutralization titers and delineate the role of the adjuvants to immunogenicity, we investigated whether sera reactivity was directed towards pre-fusion specific antigenic site Ø on DS-Cav1 or antigenic site II that is present in both pre-and post-fusion forms of RSV F protein (Fig 2A). We explored site-specific immune response with the aid of a) DS-Cav1 probes with KO mutations in antigenic site Ø, and b) DS-Cav1 and Post F probes with KO mutations in antigenic site II [12, 40]. We initially measured binding of week 5 sera to DS-Cav1, Post F, DS-Cav1 site Ø KO, DS-Cav1 site II KO and Post F site II KO probes using BioLayer Interferometry. Sera reactivity for five groups corresponding to Poly (I:C), Poly (IC:LC), SAS, SAS + Carbopol and Adjuplex showed the greatest response to DS-Cav1, followed by AddaVax and Alum. Sera corresponding to MPLA, and MPLA plus Alum exhibited the lowest binding response (Fig 2B). Furthermore, sera from all groups exhibited reduced binding to DS-Cav1 site Ø KO (Fig 2B). The pre-fusion specific site Ø KO probe bound significantly less to sera from Poly (I:C), Poly (IC:LC), SAS, SAS + Carbopol, and Adjuplex adjuvant groups relative to DS-Cav1 (*p* = 0.0006, 0.0040, 0.0043,0.0040 and 0.0019 respectively), demonstrating that these five adjuvants could facilitate a robust response to antigenic site Ø. In contrast, no significant difference was observed in sera from the Alum, MPLA, MPLA + Alum and AddaVax groups (Fig 2B), indicating that these adjuvants were unable to enhance the immune response to antigenic site Ø. Examining the sera response to antigenic sites as probed by DS-Cav1 site II knockout and Post F site II knockout probes, further demonstrated that the neutralizing antibody response was focused to pre-fusion specific antigenic sites displayed on RSV F.

### RSV F DS-Cav1-adjuvant formulations elicit antibodies of different IgG subclasses

**Fig 3:**
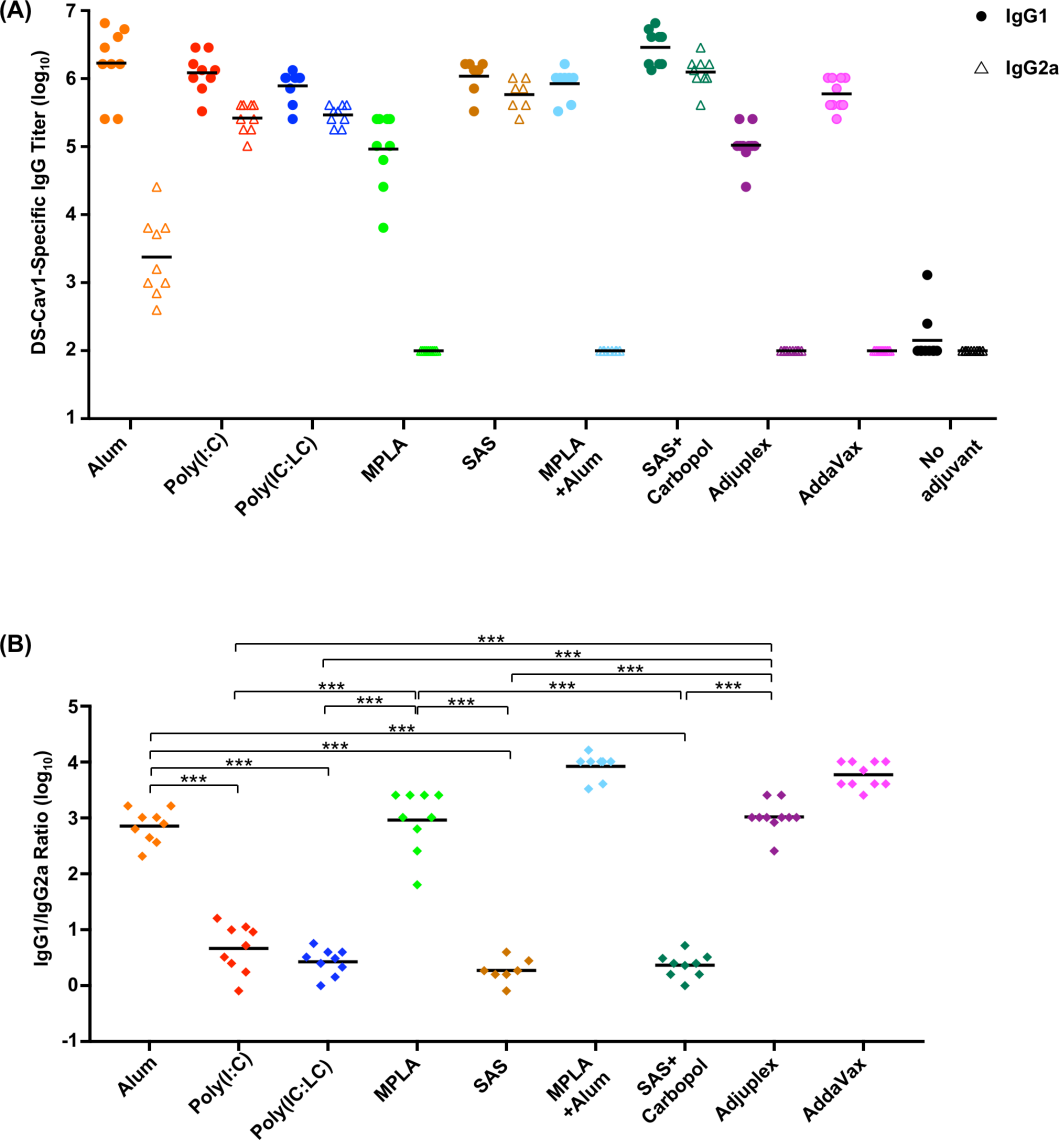
DS-Cav1-adjuvant formulations elicit antibodies of different IgG subclass in immunized mice. **(A)** Sera from naïve mice primed and boosted with DS-Cav1 adjuvant mixtures were assayed by ELISA for their RSV F DS-Cav1 specific IgG1 and IgG2a antibody responses. Scatter plots show the geometric mean, closed circles and open triangles represent IgG1 and IgG2a antibody titers, respectively. (B) The ratios of IgG1 to IgG2a for each animal in the adjuvanted groups were calculated from the upper panel. *p*-values were determined using a two-tailed Mann-Whitney test and were adjusted for multiple comparisons using the Holm-Bonferroni method, *p* < 0.001 (***). Associated raw data is listed in S3 Table.

We next analyzed sera for the quality of humoral response induced by the different adjuvant formulations and found that they elicit different IgG subclass response patterns (Fig 3). Formulations with MPLA, Alum plus MPLA, AddaVax and Adjuplex induced an IgG1 response with no detectable IgG2a response. On the other hand, DS-Cav1 formulated with Alum induced a primarily IgG1 response and DS-Cav1 formulations with SAS, SAS + Carbopol, Poly (I:C) and Poly (IC: LC) induced both an IgG1 and IgG2a response. Unadjuvanted DS-Cav1 induced a marginal IgG1 response and no detectable IgG2a response. The IgG subclass response pattern appeared to be determined not only by the properties of the adjuvant but also by the protein adjuvant formulation. The IgG2a and IgG1 subclass responses reflect the respective T helper 1 cell (Th1) and T helper 2 cell (Th2) biased immune response patterns in mice [42]. The slight IgG1 dominant response elicited by unadjuvanted DS-Cav1 is likely due to stimulation of Th2 biased response in mice [41, 43]. Although MPLA is known to augment a Th1 biased response the MPLA and MPLA + Alum adjuvant formulations were not sufficient to influence the IgG response towards a Th1 phenotype nor were Adjuplex and AddaVax sufficient to influence the IgG response towards the expected balanced Th1/Th2 response [19, 44]. As expected, the Alum adjuvant formulations stimulated primarily the expected IgG1 with some IgG2a response for Alum alone. The IgG1 to IgG2a ratio indicated a significantly greater Th2-biased response for DS-Cav1 adjuvanted with Alum, MPLA, Adjuplex (*p* < 0.001) than for Poly (I:C), Poly (IC:LC), SAS and SAS + Carbopol which gave a balanced IgG1 to IgG2a response (Fig 3A and 3B).

### Effect of RSV F DS-Cav1-adjuvant formulations on neutralization titers in elderly mice

**Fig 4:**
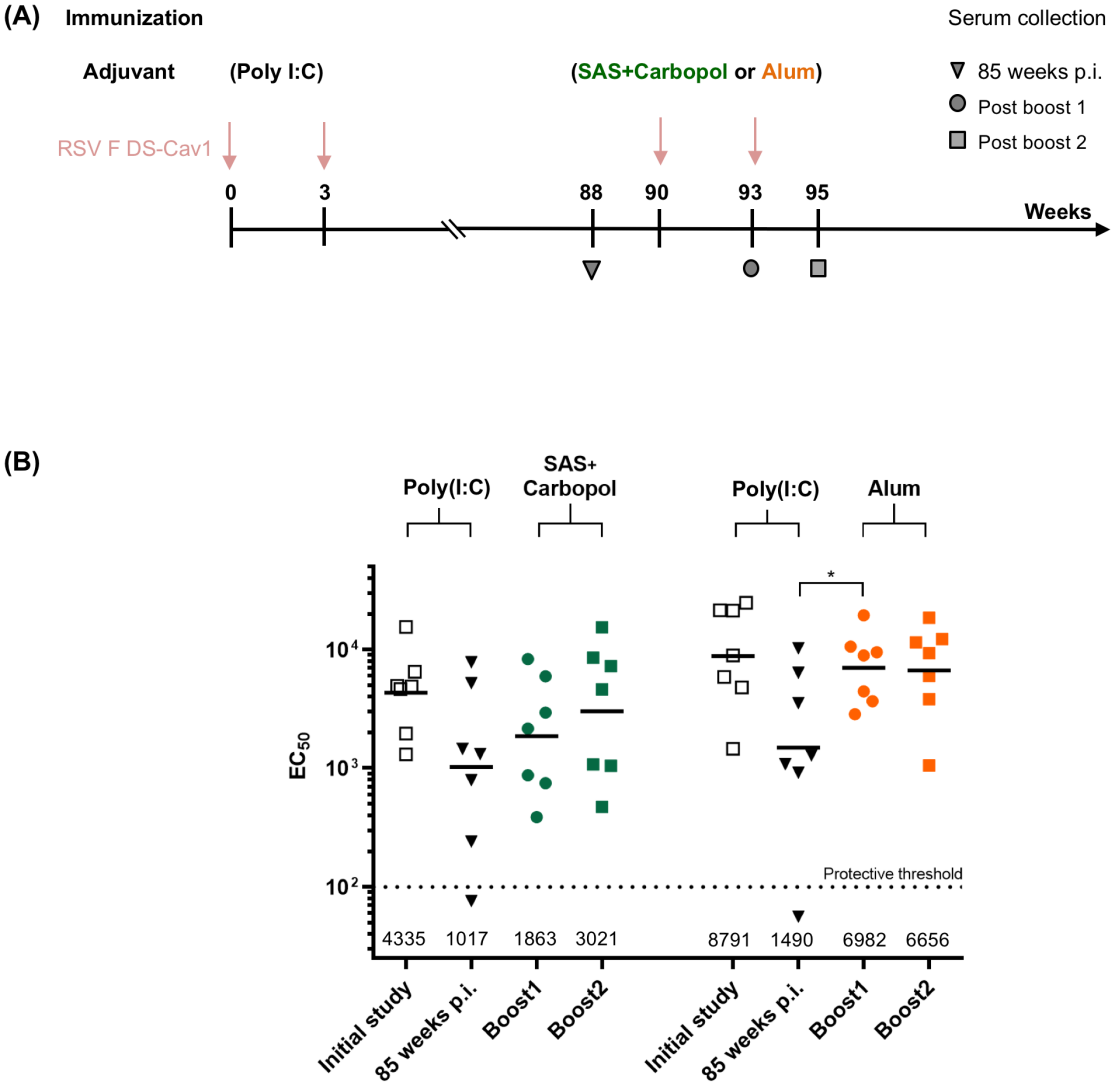
Adjuvanted DS-Cav1 can augment RSV-neutralization titers in elderly mice. **(A)** Pre-immunized elderly mice (DS-Cav1 adjuvanted with Poly (I:C), ~85 weeks wait) were administered DS-Cav1 formulated with Alum or SAS + Carbopol and the immune response for all mice assessed. **(B)** Neutralization titers for each group of seven elderly mice are shown. Scatter plots show the geometric mean (numerical value below), the palivizumab protective threshold is indicated by a dotted line [10]. *p* values were determined by two-tailed Wilcoxon matched-pairs signed rank test, *p =* >0.05 (ns); *p* < 0.05 (*). Associated raw data is reported in S4 Table.

DS-Cav1-adjuvant mixtures were used to immunize two groups of elderly CB6F1/J mice. Each group of elderly mice had been pre-immunized prior to 12 weeks of age with DS-Cav1-Poly (I:C) formulation and were administered two final DS-Cav1 boosts at week 90 and 93 respectively formulated with Alum or SAS + Carbopol after a waiting period of ~85 weeks (Fig 4A) and their immune responses were assessed (Fig 4B). Reciprocal EC_50_ neutralization titers had waned during the ~85 week interval period. However, a single boost with DS-Cav1 with Alum formulation increased the neutralization titers to the week five level (*p* = 0.0313) and titers did not increase after a second immunization at week 93. Interestingly, when DS-Cav1 adjuvanted with SAS + Carbopol was used to boost the elderly mice, neutralization titers increased from the waned response (*p =* >0.05), but remained lower than the initial response observed in young mice. Thus, in elderly mice that experienced multiple RSV immunizations as young mice, but still had modest levels of serum-neutralizing activity, boosting the response with alum as an adjuvant could effectively achieve above threshold levels of protective immunity.

### RSV F DS-Cav1-adjuvant formulations direct the antibody response to antigenic sites Ø and II in elderly mice

**Fig 5:**
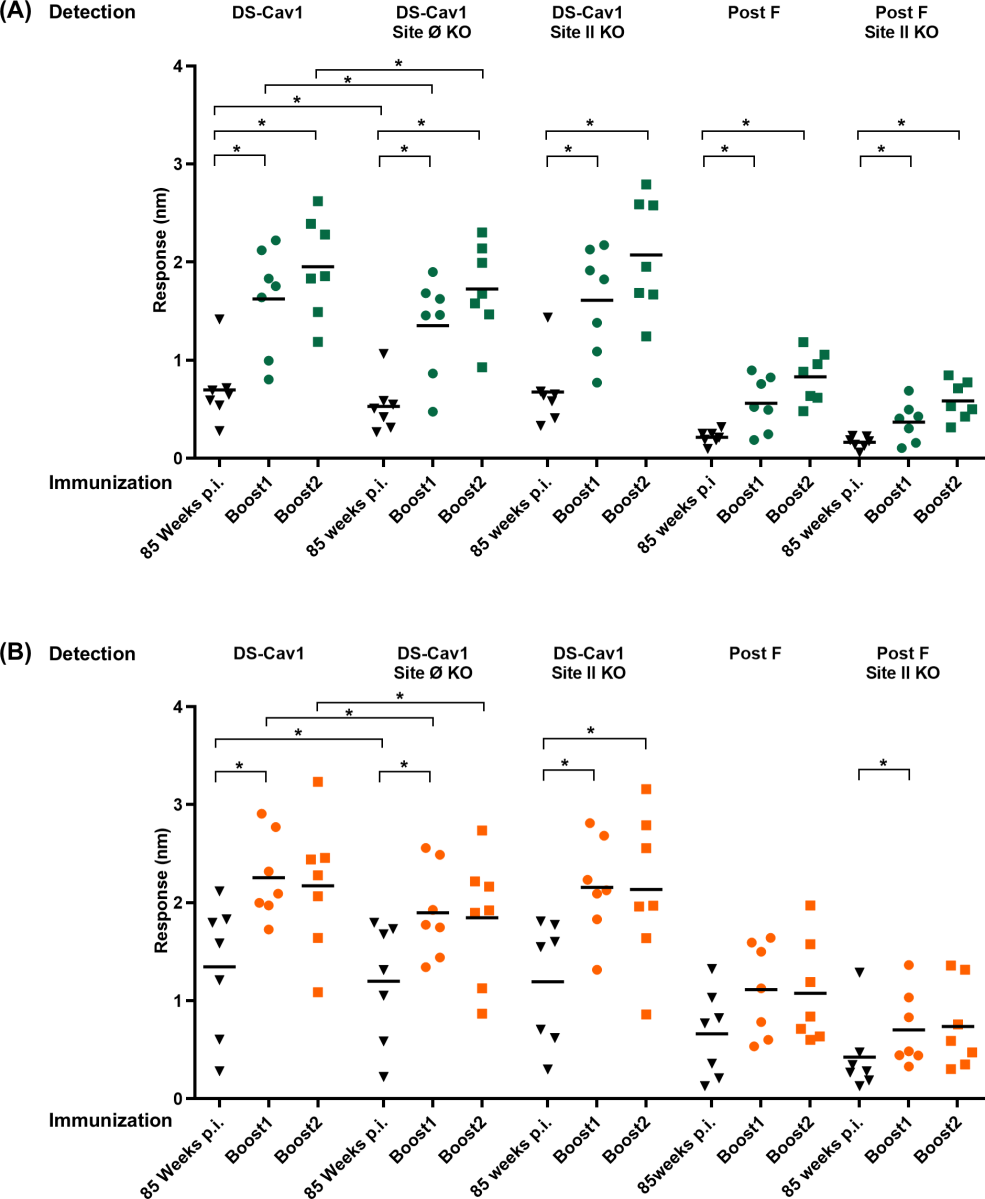
Boost by adjuvanted RSV F DS-Cav1 can focus the RSV immune response to antigenic site Ø and II in elderly mice. Pre-immunized elderly mice (~85 weeks wait) were administered **(A)** DS-Cav1 formulated with SAS + Carbopol or **(B)** DS-Cav1 formulated with Alum and recognition of DS-Cav1, DS-Cav1 site Ø KO, DS-Cav1 site II KO, Post F and Post F site II KO probes by sera for all immunized mice assessed. Scatter plots show the geometric mean. *p* values were determined by two-tailed Wilcoxon matched-pairs signed rank test, *p* = >0.05 (ns); *p* < 0.05 (*). Associated raw data is reported in S5 Table.

We further investigated whether sera reactivity in pre-immunized elderly mice was directed towards pre-fusion specific antigenic site Ø on DS-Cav1 or antigenic site II that is present in both pre- and post-fusion forms of the RSV F protein (Fig 5). As in the case of young mice, we explored site-specific immune responses with the aid of a) DS-Cav1 probes with KO mutations in antigenic site Ø and b) DS-Cav1 and Post F probes with KO mutations in antigenic site II [12, 40]. We measured binding of sera from ~85 weeks post immunization as well as the two boosts to DS-Cav1, Post F, DS-Cav1 site Ø KO, DS-Cav1 site II KO and Post F site II KO probes using BLI. Sera responses to the pre- and post- F probes exhibited a profile nearly identical to the neutralization titers (Figs 4 and 5) and we also observed a significant fraction of response (*p =* <0.05) targeted to antigenic site Ø.

### RSV F DS-Cav1-adjuvant formulations modulate antibody responses in elderly mice

**Fig 6:**
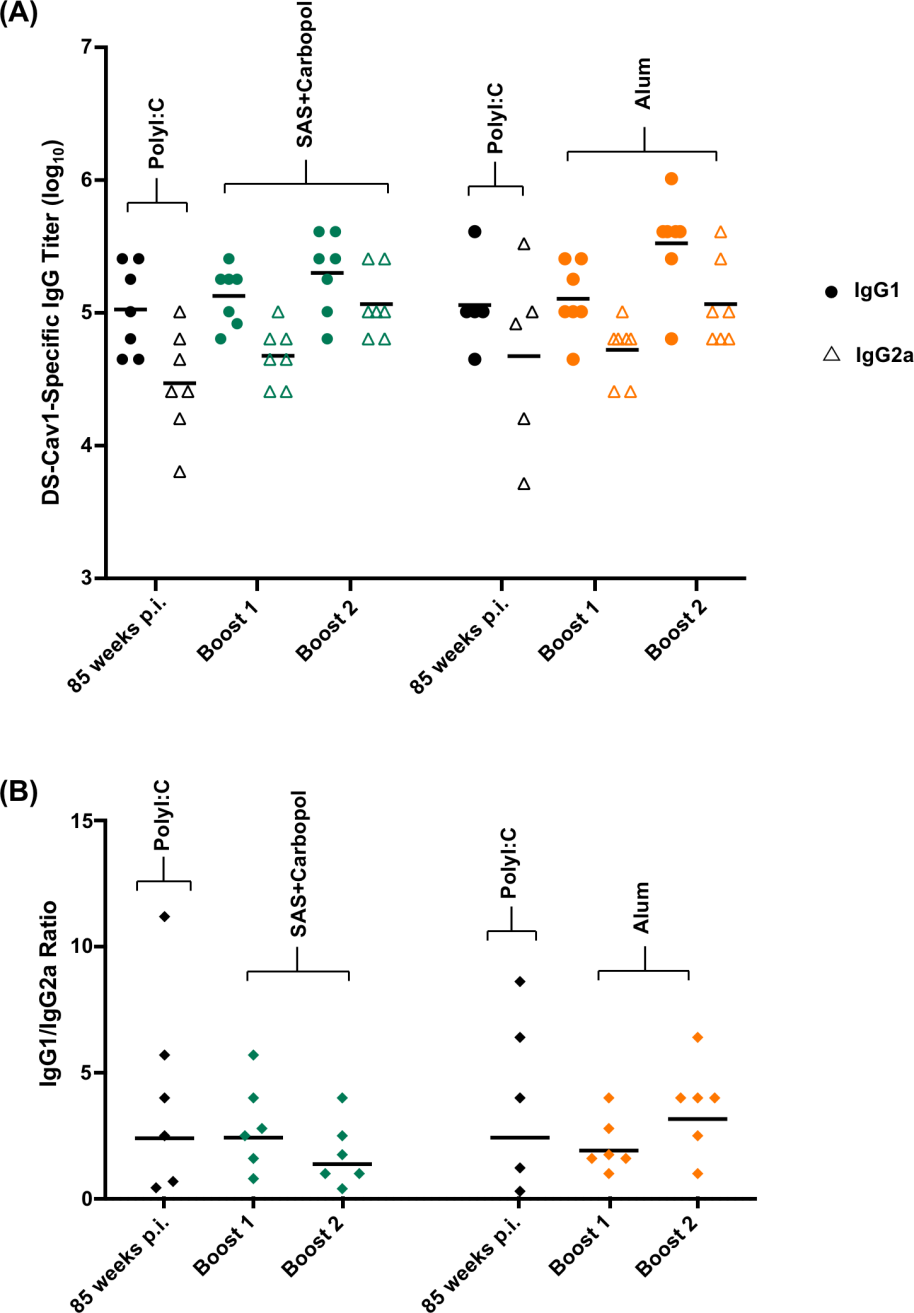
Prime-boost vaccination with different adjuvant formulations induced a balanced Th1/Th2 IgG response in elderly mice. **(A**) Pre-immunized elderly mice (~85 weeks wait) were administered DS-Cav1 formulated with SAS + Carbopol or Alum. Sera were assayed by ELISA for their DS-Cav1 specific IgG1 and IgG2a antibody responses. Closed circles and open triangles represent IgG1 and IgG2a antibody titers, respectively. **(B**) The ratios of IgG1 to IgG2a for each animal in the groups were calculated from the upper panel. *p* values were determined by two-tailed Wilcoxon matched-pairs signed rank test, *p =* >0.05 (ns). Associated raw data is listed in S6 Table.

We also analyzed sera from the elderly mice immunizations for the quality of humoral response induced by Alum and SAS + Carbopol adjuvant formulations (Fig 6A and 6B). Interestingly, both groups of DS-Cav1 adjuvant formulations elicited a balanced IgG1 and IgG2a subclass response pattern. As expected, elderly mice immunized with DS-Cav1 adjuvanted with SAS + Carbopol, retained the IgG subclass response pattern observed in young mice (Figs 3A and 6A). On the other hand, elderly mice immunized with alum-adjuvanted DS-Cav1 did not elicit a strongly IgG1 skewed response as observed in young mice, but instead elicited a more balanced IgG2a/IgG1 response. The IgG1 to IgG2a ratios of boost 1 and boost 2 in the SAS + Carbopol and Alum groups were not significantly different from their corresponding time point at 85 weeks p.i. (*p* = >0.05). Thus, the IgG subclass response pattern appeared to be determined by the priming immunization as the DS-Cav1-Poly (I:C) adjuvant formulation induced a balanced IgG1/IgG2a response in naïve mice.

### Effect of adjuvant formulations on neutralization titers of bovine RSV F DS-Cav1 in mice and cattle

**Fig 7:**
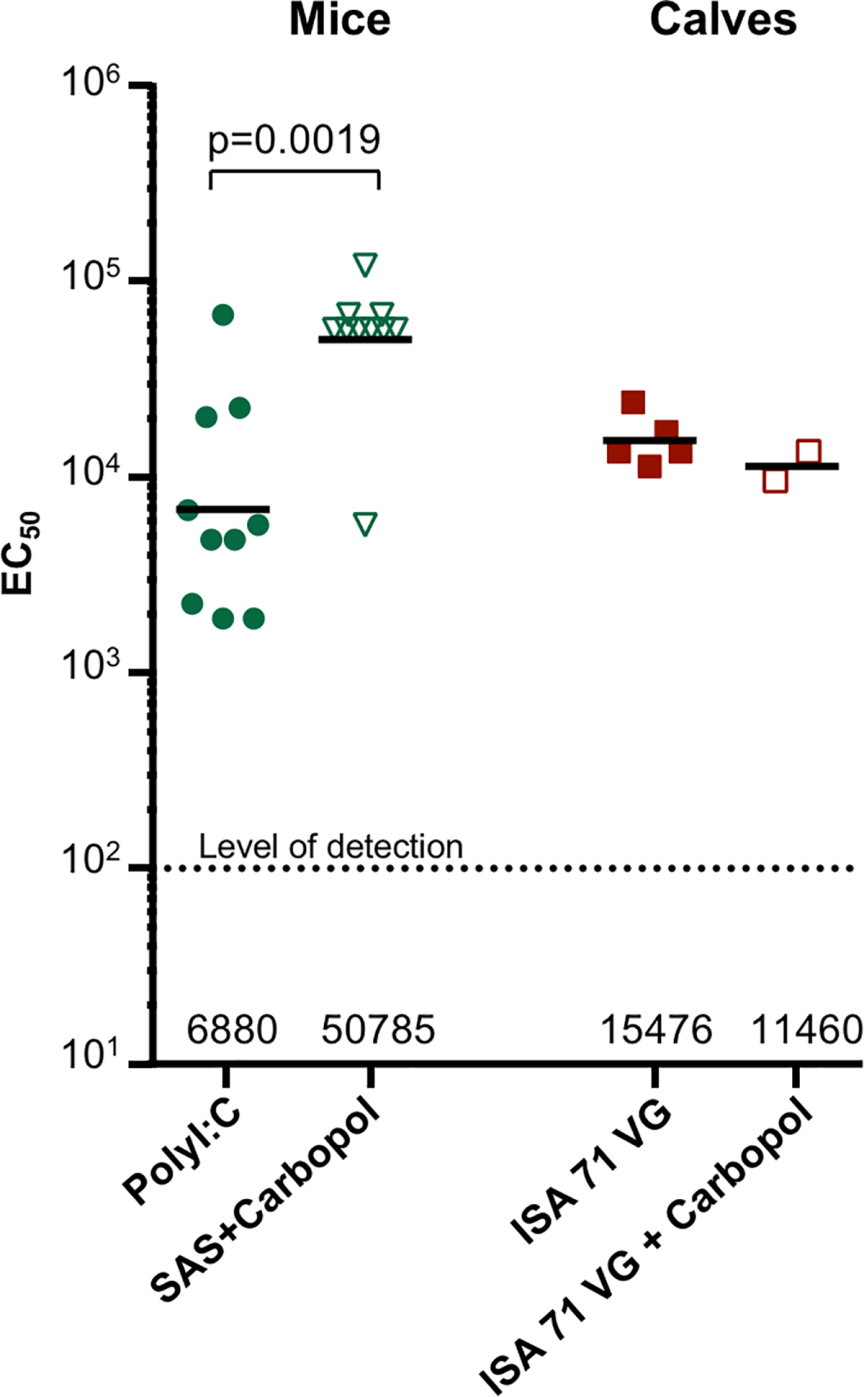
Bovine pre-fusion RSV F response in mice and cattle. Pre-F bovine RSV F DS-Cav1 formulated with two adjuvants was used to immunize mice and calves and resulted in high neutralization titers. Mice were administered DS-Cav1 formulated with Poly(I:C) and SAS + Carbopol, whereas, cattle were administered with an analogous water in oil emulsion, ISA 71 VG-DS-Cav1 formulation and ISA 71 VG + Carbopol–DS-Cav1 formulation. Scatter plots show the geometric mean (numerical value below), each group included 10 mice and 2–5 calves. Level of detection is indicated by a dotted line; and *p* value for SAS + Carbopol/DS-Cav1 versus the Poly (I:C) formulations in mice as assessed by two-tailed Mann-Whitney test is shown; *p* = 0.0019. (Control groups without Carbopol were reported previously in ref. [36]).

We tested whether the very high EC_50_ titers observed with DS-Cav1 adjuvanted SAS + Carbopol in mice were also higher with pre-F stabilized bovine RSV F in mice and cattle. Bovine DS-Cav1 adjuvanted with Poly (I:C) and SAS + Carbopol mixtures were used to immunize two groups of 10 CB6F1/J mice. After two immunizations, in the week five sera, significantly higher reciprocal EC_50_ neutralization titers were observed in the SAS + Carbopol group as compared to the Poly (I:C) group (with geometric mean titers of 50784 and 6879, respectively, and a *p* value = 0.0019, Fig 7). We next immunized cattle with bovine DS-Cav1 adjuvanted with the analogous water in oil adjuvant ISA 71 VG and ISA 71 VG-Carbopol formulations. Rather surprisingly, no significant difference was observed in neutralization titers from the ISA 71 VG, and ISA 71 VG plus Carbopol groups indicating this combination of adjuvants did not significantly enhance the immune response in cattle (Fig 7).

## Discussion

Vaccine formulations that include antigens and agonists of the immune system and antigen presenting cells that selectively present a target antigen to the immune system can greatly influence the immunity elicited from vaccination. Adjuvants may influence the type of immune profile as well as the breadth of neutralizing antibodies [45] or the persistence of the protective response. One of the major goals of vaccination against RSV is to induce immunity in a wide range of age groups from neonates to the elderly. In the elderly population who have experienced repeated RSV infections throughout life, and have some pre-existing immunity, boosting the response to neutralization-sensitive epitopes could achieve threshold levels of protective immunity. This may require formulations of a RSV vaccine with different adjuvants. We undertook a comparative study of adjuvants on a single immunogen RSV F DS-Cav1 in naïve and elderly CB6F1/J mice. In our study with naïve mice significantly higher EC_50_ neutralization titers were observed in six groups of mice immunized with DS-Cav1: Alum, SAS, SAS + Carbopol, Adjuplex, Poly (I:C) and Poly (IC:LC) than for DS-Cav1 adjuvanted with TLR4 agonist MPLA, Alum plus MPLA and AddaVax, whereas unadjuvanted DS-Cav1 titers were below the protective threshold (Fig 1). Alum surprisingly exhibited stronger adjuvant effects on the magnitude of antibody response than MPLA or Alum + MPLA when formulated with DS-Cav1, even though ASO4 (Alum plus MPLA formulation) has a synergistic effect when adjuvanted with HPV virus-like particles [46]. Thus, immunogenicity may be influenced not just by the properties and concentration or dose of the adjuvant [47] but also by the combination of immunogen and adjuvant. It is not known whether there is a specific property of Alum that improves the response or whether TLR4 agonists like MPLA in some way interfere with the responses to RSV F.

Titers of pre-fusion-F specific antibodies have been shown to correlate with the magnitude of RSV neutralizing activity in human sera [40]. We therefore interrogated DS-Cav1- immune sera using three antigenic site-specific probes (Fig 2B) and found that as with human sera, highly neutralizing sera from Poly (I:C), Poly (IC:LC), SAS and SAS + Carbopol groups elicit polyclonal response primarily to site Ø and to a much lesser extent to site II presented in the pre-fusion state of RSV F.

IgG2a and IgG1 subclass responses, reflect respective T helper 1 (Th1) and T helper 2 cell (Th2)-biased immune response patterns in mice [42]. Interestingly, SAS, SAS + Carbopol, Poly (I:C) and Poly (IC:LC) groups induced a balanced Th1/Th2 IgG response, whereas Alum, MPLA, Alum plus MPLA, Adjuplex and Addavax induced a predominantly Th2-biased IgG1 response. Adjuvant groups with high neutralization titers against RSV exhibited a balanced Th1/Th2 response. High titer neutralizing antibodies have been reported with a balanced Th1/Th2 response when mice were immunized with reconstituted virosomes produced from RSV envelopes containing a lipopeptide adjuvant [48]. In contrast, to the Th2-biased response induced by DS-Cav1 with Adjuplex, a balanced Th1/Th2 response was obtained when mice were immunized with influenza HA protein formulated with Adjuplex [19]. In elderly mice with some pre-existing immunity, boosting the response with Alum as an adjuvant could achieve high levels of protective immunity whereas with SAS + Carbopol as an adjuvant, more than one immunization was necessary to achieve a similar level of neutralization. In contrast, Alum adjuvant with RSV F protein in elderly BALB/c mice also conferred protection (neutralizing anti-RSV F antibody titers) albeit the protection was sub-optimal compared to that seen in young mice [49]. Interestingly, in the Alum group for elderly mice, the IgG1/IgG2a response was determined by the priming immunization of naïve mice as the DS-Cav1-Poly (I:C) prime boost regimen induced a balanced IgG1/IgG2a pattern. Thus, our results indicate that the RSV F vaccine immune response in mice is not just influenced by the properties and concentration or dose of the adjuvant [47] but can be augmented by combinations of antigen and adjuvant and adjuvants that enable a Th1/Th2 –balanced response result in higher RSV neutralizing activity in mice. Overall, our data support the fact that adjuvants play less of a role in determining responses following a boost in pre-immune animals than they do in the inductive event following a primary immunization. While the addition of Carbopol to the oil-in-water adjuvant SAS enhanced the antibody response in mice, the addition of Carbopol to the water-in-oil adjuvant ISA 71 VG did not appear to enhance the antibody response in calves. Overall, our results suggest that the particulars of different adjuvants may be species specific. Furthermore, the role of adjuvants in the context of RSV immunization and the legacy of formalin-inactivated RSV (FI-RSV) vaccine-enhanced illness should be considered. There are two major immunological patterns associated with the formalin-inactivated RSV (FI-RSV) vaccine-enhanced illness. One was poor functional neutralizing antibody (NT) activity despite induction of significant binding antibody. This is thought to contribute to immune complex deposition in small airways that were documented in the children who died from the FI-RSV enhanced respiratory disease (ERD) [50]. The second pattern was a Th2-biased CD4 T cell response associated with eosinophilia and neutrophilic alveolitis [51]. Although it is possible that if sufficient NT activity is induced and the specificity of the response is corrected that controlling the T cell response patterns with adjuvants may make it possible to test protein-based vaccines again in RSV-naïve neonates, it will be a difficult regulatory hurdle. If the dose of a stabilized pre-F antigen is reduced to very low levels to induce a subprotective antibody response, it is possible to simulate the pattern of lung inflammation seen with full doses of less effective vaccines [52]. Therefore, just using the correct conformation of a vaccine antigen and redirecting antibody to neutralization-sensitive sites may not be sufficient, even with Th1-biased adjuvants, to prevent a pathogenic inflammatory response to breakthrough infection. For the pre-F vaccine antigen delivered as protein the ultimate target populations are pregnant women, the elderly, and potentially older children. All these groups will have been primed by natural infection, so the role of adjuvant is primarily to increase the magnitude of the response based on the established precursor T cell and B cell populations. For pregnant women, vaccination should avoid excessive Th1-biased responses for safety concerns related to the pregnancy. If adjuvants are needed to boost antibody responses to protective levels in adults, alum-based formulations may have an acceptable safety and immunological profile for use in pregnancy. In the elderly or older children, the most potent, tolerable adjuvant should be chosen based on results from early phase clinical trials.

## Supporting information

S1 Table Adjuvants augment pre-F RSV F response in mice. Neutralization titers for the 10 groups of mice are listed.
(DOCX)

S2 Table Sera analysis of mice immunized with DS-Cav1 adjuvant formulations. BLI analysis using DS-Cav1 site specific KO probes.
(DOCX)

S3 Table ELISA measurement of IgG response in immunized mice. (DOCX)

S4 Table Neutralization titers in elderly mice. (DOCX)

S5 Table Elderly mice sera analysis using DS-Cav1 site specific KO probes. BLI analysis using DS-Cav1 site specific KO probes.
(DOCX)

S6 Table ELISA measurement of IgG response in elderly mice. (DOCX)
