# Supplementary material for: Adjuvants and the vaccine response to the DS-Cav1-stabilized fusion glycoprotein of respiratory syncytial virus
Source: PLoS One. 2017 Oct 26;12(10):e0186854. doi: 10.1371/journal.pone.0186854 (PMC5658087; doi:10.1371/journal.pone.0186854)
Supplement: S1 Table — Neutralization titers for the 10 groups of mice are listed. (DOCX) [file pone.0186854.s001.docx]

**S1 Table. Adjuvants augment pre-F RSV F response in mice.**

| **Animal**  **number** | **Poly (I:C)** | **Animal**  **number** | **Poly (IC:LC)** | **Animal**  **number** | **Alum** | **Animal**  **number** | **SAS** | **Animal**  **number** | **SAS+ Carbopol** | **Animal**  **number** | **Alum+ MPLA** | **Animal**  **number** | **MPLA** | **Animal**  **number** | **AddaVax** | **Animal**  **number** | **Adjuplex** | **Animal**  **number** | **No adjuvant** |
| --- | --- | --- | --- | --- | --- | --- | --- | --- | --- | --- | --- | --- | --- | --- | --- | --- | --- | --- | --- |
| 3791 | 2552 | 3801 | 2756 | 3811 | 2021 | 3821 | 10204 | 3831 | 49202 | 566 | 1213 | 576 | 1872 | 586 | 1332 | 596 | 4510 | 606 | 5 |
| 3792 | 8434 | 3802 | 2918 | 3812 | 9224 | 3822 | 1418 | 3832 | 15095 | 567 | 905 | 577 | 617 | 587 | 1953 | 597 | 8721 | 607 | 5 |
| 3793 | 1170 | 3803 | 2843 | 3813 | 7910 | 3823 | 3328 | 3833 | 26925 | 568 | 1609 | 578 | 2154 | 588 | 307 | 598 | 1928 | 608 | 5 |
| 3794 | 2248 | 3804 | 8024 | 3814 | 6108 | 3824 | 15372 | 3834 | 13666 | 569 | 1637 | 579 | 866 | 589 | 1105 | 599 | 1725 | 609 | 5 |
| 3795 | 13046 | 3805 | 8476 | 3815 | 1455 | 3825 | 6788 | 3835 | 18475 | 570 | 554 | 580 | 2762 | 590 | 2182 | 600 | 10957 | 610 | 5 |
| 3796 | 4954 | 3806 | 12048 | 3816 | 2773 | 3826 | 22667 | 3836 | 6838 | 571 | 3919 | 581 | 1062 | 591 | 513 | 601 | 5108 | 611 | 5 |
| 3798 | 3232 | 3807 | 2493 | 3817 | 7212 | 3827 | 8178 | 3837 | 9235 | 572 | 650 | 582 | 2660 | 592 | 166 | 602 | 2511 | 612 | 5 |
| 3799 | 1555 | 3808 | 1503 | 3818 | 17804 | 3828 | 6406 | 3838 | 77482 | 573 | 1685 | 583 | 8573 | 593 | 12051 | 603 | 5630 | 613 | 5 |
| 3800 | 9304 | 3809 | 2324 | 3819 | 1512 | 3829 | 4049 | 3839 | 5657 | 574 | 1125 | 584 | 3687 | 594 | 1736 | 604 | 17679 | 614 | 5 |
| 3801 | 2713 | 3810 | 2632 | 3820 | 10213 | 3830 | 13631 | 3840 | 44020 | 575 | 1216 | 585 | 3598 | 595 | 3028 | 605 | 6399 | 615 | 14 |
