# Supplementary material for: Adjuvants and the vaccine response to the DS-Cav1-stabilized fusion glycoprotein of respiratory syncytial virus
Source: PLoS One. 2017 Oct 26;12(10):e0186854. doi: 10.1371/journal.pone.0186854 (PMC5658087; doi:10.1371/journal.pone.0186854)
Supplement: S2 Table — BLI analysis using DS-Cav1 site specific KO probes. (DOCX) [file pone.0186854.s002.docx]

**S2 Table.** **Sera analysis of mice immunized with DS-Cav1 adjuvant formulations.**

| **Animal number**  **(Alum)** | **DS-Cav1** | | **DS-Cav1**  **Site Ø KO** | **DS-Cav1**  **Site II KO** | | **Post** | | | **Post**  **Site II KO** |
| --- | --- | --- | --- | --- | --- | --- | --- | --- | --- |
| 3811 | 2.22 | | 1.97 | 2.33 | | 1.41 | | | 0.87 |
| 3812 | 1.78 | | 1.59 | 1.87 | | 1.39 | | | 1.11 |
| 3813 | 1.83 | | 1.66 | 1.87 | | 1.38 | | | 1.09 |
| 3814 | 0.61 | | 0.48 | 0.61 | | 0.30 | | | 0.25 |
| 3815 | 1.92 | | 1.75 | 2.24 | | 1.37 | | | 1.26 |
| 3816 | 1.50 | | 1.42 | 1.75 | | 0.87 | | | 0.83 |
| 3817 | 1.85 | | 1.56 | 1.93 | | 1.00 | | | 0.75 |
| 3818 | 1.57 | | 1.32 | 1.71 | | 1.21 | | | 1.02 |
| 3819 | 0.99 | | 0.85 | 1.08 | | 0.73 | | | 0.55 |
| 3820 | 1.52 | | 1.43 | 1.74 | | 1.11 | | | 0.86 |
| **Animal number**  **(Poly (I:C))** | **DS-Cav1** | | **DS-Cav1**  **Site Ø KO** | **DS-cav1**  **Site II KO** | | **Post** | | | **Post**  **Site II KO** |
| 3791 | 2.13 | | 1.80 | 2.17 | | 1.16 | | | 0.84 |
| 3792 | 2.02 | | 1.75 | 2.12 | | 1.17 | | | 0.89 |
| 3794 | 2.29 | | 1.90 | 2.18 | | 0.92 | | | 0.76 |
| 3795 | 2.03 | | 1.75 | 2.07 | | 1.10 | | | 0.88 |
| 3796 | 1.95 | | 1.53 | 2.23 | | 1.03 | | | 0.93 |
| 3799 | 1.90 | | 1.67 | 2.08 | | 0.99 | | | 0.63 |
| 3800 | 1.96 | | 1.91 | 2.16 | | 0.99 | | | 0.81 |
| 3801 | 1.92 | | 1.73 | 2.04 | | 0.78 | | | 0.69 |
| **Animal number**  **(Poly (IC:LC))** | **DS-Cav1** | | **DS-Cav1**  **Site Ø KO** | **DS-Cav1**  **Site II KO** | | **Post** | | | **Post**  **Site II KO** |
| 3801 | 2.23 | | 1.92 | 2.35 | | 1.09 | | | 0.77 |
| 3802 | 2.17 | | 1.87 | 2.23 | | 0.85 | | | 0.64 |
| 3803 | 1.75 | | 1.53 | 1.85 | | 0.83 | | | 0.51 |
| 3804 | 2.12 | | 1.67 | 1.97 | | 0.78 | | | 0.56 |
| 3805 | 1.86 | | 1.62 | 2.00 | | 0.79 | | | 0.55 |
| 3806 | 2.11 | | 1.64 | 2.07 | | 1.16 | | | 0.91 |
| 3808 | 1.98 | | 1.75 | 2.02 | | 0.96 | | | 0.70 |
| 3809 | 1.74 | | 1.47 | 1.78 | | 0.72 | | | 0.47 |
| 3810 | 1.91 | | 1.68 | 1.98 | | 0.65 | | | 0.53 |
| **Animal number**  **(MPLA)** | **DS-Cav1** | | **DS-Cav1**  **Site Ø KO** | **DS-Cav1**  **Site II KO** | | **Post** | | | **Post**  **Site II KO** |
| 576 | 1.05 | | 0.83 | 1.06 | | 0.63 | | | 0.44 |
| 578 | 1.17 | | 1.12 | 1.35 | | 0.62 | | | 0.48 |
| 581 | 1.11 | | 0.91 | 1.20 | | 0.57 | | | 0.41 |
| 582 | 0.89 | | 0.69 | 0.83 | | 0.52 | | | 0.27 |
| 583 | 1.44 | | 1.11 | 1.58 | | 0.89 | | | 0.66 |
| 584 | 1.73 | | 1.54 | 1.77 | | 0.93 | | | 0.63 |
| 585 | 0.40 | | 0.32 | 0.37 | | 0.36 | | | 0.34 |
| **Animal number**  **(SAS)** | **DS-Cav1** | | **DS-Cav1**  **Site Ø KO** | **DS-Cav1**  **Site II KO** | | **Post** | | | **Post**  **Site II KO** |
| 3821 | 2.29 | | 1.91 | 2.34 | | 1.45 | | | 0.94 |
| 3822 | 2.25 | | 1.90 | 2.29 | | 1.39 | | | 1.09 |
| 3823 | 1.95 | | 1.57 | 2.09 | | 0.99 | | | 0.69 |
| 3825 | 1.90 | | 1.62 | 2.05 | | 1.31 | | | 1.09 |
| 3826 | 1.96 | | 1.68 | 2.26 | | 1.19 | | | 1.12 |
| 3829 | 2.13 | | 1.84 | 2.19 | | 1.37 | | | 1.09 |
| **Animal number**  **(MPLA + Alum)** | **DS-Cav1** | | **DS-Cav1**  **Site Ø KO** | **DS-Cav1**  **Site II KO** | | **Post** | | | **Post**  **Site II KO** |
| 566 | 1.39 | | 0.92 | 1.29 | | 1.07 | | | 0.91 |
| 567 | 1.48 | | 1.27 | 1.53 | | 1.20 | | | 0.91 |
| 568 | 1.60 | | 1.37 | 1.39 | | 1.17 | | | 0.73 |
| 570 | 1.50 | | 1.36 | 1.56 | | 1.19 | | | 0.97 |
| 571 | 1.98 | | 1.55 | 2.13 | | 1.63 | | | 1.43 |
| 572 | 1.24 | | 1.17 | 1.39 | | 0.94 | | | 0.79 |
| 574 | 1.41 | | 1.13 | 1.44 | | 1.06 | | | 0.87 |
| 575 | 1.03 | | 0.83 | 0.96 | | 1.10 | | | 1.02 |
| **Animal number**  **(SAS + Carbopol)** | **DS-Cav1** | **DS-Cav1**  **Site Ø KO** | | | **DS-Cav1**  **Site II KO** | | **Post** | **Post**  **Site II KO** | |
| 3831 | 2.29 | 1.82 | | | 2.37 | | 1.71 | 1.21 | |
| 3832 | 2.31 | 1.86 | | | 2.29 | | 1.50 | 0.91 | |
| 3833 | 2.12 | 1.92 | | | 2.33 | | 1.31 | 0.87 | |
| 3835 | 2.31 | 2.02 | | | 2.48 | | 1.57 | 1.20 | |
| 3836 | 2.19 | 1.82 | | | 2.26 | | 1.49 | 1.41 | |
| 3837 | 2.30 | 1.73 | | | 2.29 | | 1.66 | 1.23 | |
| 3838 | 2.16 | 1.87 | | | 2.31 | | 1.49 | 1.14 | |
| 3839 | 1.59 | 1.68 | | | 1.82 | | 1.49 | 1.05 | |
| 3840 | 2.19 | 1.99 | | | 2.25 | | 1.36 | 1.19 | |
| **Animal number**  **(Adjuplex)** | **DS-Cav1** | **DS-Cav1**  **Site Ø KO** | | | **DS-Cav1**  **Site II KO** | | **Post** | **Post**  **Site II KO** | |
| 596 | 2.11 | 1.73 | | | 2.10 | | 1.25 | 0.86 | |
| 598 | 1.81 | 1.53 | | | 1.80 | | 0.98 | 0.62 | |
| 599 | 1.69 | 1.20 | | | 1.49 | | 0.85 | 0.50 | |
| 601 | 2.30 | 1.69 | | | 2.29 | | 1.41 | 1.04 | |
| 602 | 2.15 | 1.63 | | | 2.12 | | 1.20 | 0.86 | |
| 603 | 1.99 | 1.70 | | | 2.03 | | 1.31 | 0.92 | |
| 604 | 2.07 | 1.75 | | | 2.09 | | 1.09 | 0.78 | |
| 605 | 2.06 | 1.80 | | | 2.03 | | 1.23 | 1.12 | |
| **Animal number**  **(AddaVax)** | **DS-Cav1** | **DS-Cav1**  **Site Ø KO** | | | **DS-Cav1**  **Site II KO** | | **Post** | **Post**  **Site II KO** | |
| 586 | 1.54 | 1.41 | | | 1.63 | | 1.22 | 1.05 | |
| 587 | 2.11 | 1.85 | | | 2.05 | | 1.74 | 1.39 | |
| 588 | 1.78 | 1.60 | | | 1.74 | | 1.01 | 0.93 | |
| 589 | 2.45 | 1.92 | | | 2.25 | | 1.45 | 1.45 | |
| 590 | 2.10 | 1.65 | | | 1.96 | | 1.07 | 1.06 | |
| 591 | 1.61 | 1.27 | | | 1.75 | | 0.90 | 0.96 | |
| 592 | 1.59 | 1.36 | | | 1.61 | | 1.04 | 0.95 | |
| 593 | 2.28 | 1.90 | | | 2.30 | | 1.39 | 1.28 | |
| 594 | 2.03 | 1.90 | | | 2.09 | | 1.28 | 1.16 | |
| 595 | 2.13 | 2.03 | | | 2.32 | | 1.29 | 1.21 | |

BLI analysis using DS-Cav1 site specific KO probes.
