# Supplementary material for: Adjuvants and the vaccine response to the DS-Cav1-stabilized fusion glycoprotein of respiratory syncytial virus
Source: PLoS One. 2017 Oct 26;12(10):e0186854. doi: 10.1371/journal.pone.0186854 (PMC5658087; doi:10.1371/journal.pone.0186854)
Supplement: S3 Table — (DOCX) [file pone.0186854.s003.docx]

**S3 Table. ELISA measurement of IgG response in immunized mice*.**

|  | **Poly(I:C)** | | **Poly(IC:LC)** | | **Alum** | | **SAS** | | **SAS/Ccarbopol** | | **MPLA+Alum** | | **MPLA** | | **AddVax** | | **Adjuplex** | | **DS-Cav1** | |
| --- | --- | --- | --- | --- | --- | --- | --- | --- | --- | --- | --- | --- | --- | --- | --- | --- | --- | --- | --- | --- |
| **IgG1** | 1024000 | 409600 | 409600 | 1638400 | 1638400 | 1638400 | 1638400 | 1024000 | 4096000 | 4096000 | 1024000 | 1024000 | 64000 | 64000 | 409600 | 409600 | 102400 | 102400 | 100 | 100 |
|  | 1638400 | 1024000 | 1024000 | 1638400 | 4096000 | 4096000 | 1638400 | 1024000 | 6553600 | 6553600 | 256000 | 409600 | 25600 | 25600 | 409600 | 409600 | 256000 | 256000 | 1000 | 1600 |
|  | 1638400 | 4096000 | 1024000 | 409600 | 256000 | 256000 | 1024000 | 409600 | 4096000 | 6553600 | 1024000 | 1024000 | 256000 | 256000 | 256000 | 256000 | 102400 | 102400 | 100 | 100 |
|  | 1024000 | 1024000 | 409600 | 409600 | 4096000 | 6553600 | N.D. | N.D. | N.D. | N.D. | N.D. | N.D. | 102400 | 102400 | 1024000 | 1024000 | 25600 | 25600 | 100 | 100 |
|  | 1638400 | 1638400 | 1024000 | 1024000 | N.D. | N.D. | 409600 | 256000 | 1638400 | 1024000 | 1024000 | 1024000 | N.D. | N.D. | 1024000 | 1024000 | 102400 | 102400 | 100 | 100 |
|  | 256000 | 409600 | 1024000 | 1024000 | 6553600 | 6553600 | 1638400 | 1638400 | 1638400 | 1638400 | 1638400 | 1638400 | 256000 | 256000 | 409600 | 409600 | 256000 | 256000 | 250 | 250 |
|  | N.D. | N.D. | N.D. | N.D. | 1638400 | 1638400 | 1638400 | 1638400 | 4096000 | 4096000 | 1024000 | 1024000 | 102400 | 102400 | 409600 | 409600 | 102400 | 102400 | 100 | 100 |
|  | 1638400 | 1024000 | 1024000 | 1024000 | 1638400 | 1638400 | 1638400 | 1638400 | 4096000 | 4096000 | N.D. | N.D. | 256000 | 256000 | 1024000 | 1024000 | 102400 | 102400 | 100 | 100 |
|  | 4096000 | 1638400 | 256000 | 256000 | 256000 | 256000 | N.D. | N.D. | 1638400 | 1638400 | 1024000 | 1024000 | 6400 | 6400 | 1024000 | 409600 | 102400 | 64000 | 100 | 100 |
|  | 1638400 | 409600 | 1024000 | 1024000 | 4096000 | 1638400 | N.D. | N.D. | 1638400 | 1638400 | 409600 | 409600 | 256000 | 256000 | 1024000 | 1024000 | 102400 | 102400 | 100 | 100 |
| **IgG2a** | 409600 | 409600 | 102400 | 256000 | 1600 | 400 | 1024000 | 409600 | 1638400 | 1638400 | 100 | 100 | 100 | 100 | 100 | 100 | 100 | 100 | 100 | 100 |
|  | 256000 | 256000 | 409600 | 409600 | 4000 | 6400 | 1024000 | 409600 | 1638400 | 4096000 | 100 | 100 | 100 | 100 | 100 | 100 | 100 | 100 | 100 | 100 |
|  | 256000 | 102400 | 256000 | 409600 | 1000 | 400 | 256000 | 256000 | 1024000 | 1024000 | 100 | 100 | 100 | 100 | 100 | 100 | 100 | 100 | 100 | 100 |
|  | 102400 | 102400 | 409600 | 409600 | 25600 | 25600 | N.D. | N.D. | N.D. | N.D. | N.D. | N.D. | 100 | 100 | 100 | 100 | 100 | 100 | 100 | 100 |
|  | 102400 | 256000 | 409600 | 409600 | N.D. | N.D. | 409600 | 409600 | 409600 | 409600 | 100 | 100 | 100 | 100 | 100 | 100 | 100 | 100 | 100 | 100 |
|  | 409600 | 409600 | 256000 | 256000 | 6400 | 6400 | 409600 | 409600 | 1024000 | 1024000 | 100 | 100 | 100 | 100 | 100 | 100 | 100 | 100 | 100 | 100 |
|  | N.D. | N.D. | N.D. | N.D. | 1600 | 400 | 1024000 | 1024000 | 1024000 | 1638400 | 100 | 100 | 100 | 100 | 100 | 100 | 100 | 100 | 100 | 100 |
|  | 409600 | 409600 | 256000 | 256000 | 1600 | 1600 | 1024000 | 1024000 | 1638400 | 1638400 | N.D. | N.D. | 100 | 100 | 100 | 100 | 100 | 100 | 100 | 100 |
|  | 256000 | 256000 | 256000 | 102400 | 400 | 400 | N.D. | N.D. | 1638400 | 1638400 | 100 | 100 | 100 | 100 | 100 | 100 | 100 | 100 | 100 | 100 |
|  | 409600 | 409600 | 409600 | 256000 | 6400 | 6400 | N.D. | N.D. | 1024000 | 1024000 | 100 | 100 | N.D. | N.D. | N.D. | N.D. | 100 | 100 | 100 | 100 |

N.D., not determined due to insufficient serum sample availability for all of the analysis.

* Animal numbers for each group are in same order as S1 Table.
