# Supplementary material for: Adjuvants and the vaccine response to the DS-Cav1-stabilized fusion glycoprotein of respiratory syncytial virus
Source: PLoS One. 2017 Oct 26;12(10):e0186854. doi: 10.1371/journal.pone.0186854 (PMC5658087; doi:10.1371/journal.pone.0186854)
Supplement: S4 Table — (DOCX) [file pone.0186854.s004.docx]

**S4 Table.** **Neutralization titers in elderly mice.**

| **A: Elderly mice pre-immunized with DS-Cav1/Poly(I:C) DS-Cav1 adjuvanted with SAS+Carbopol** | | | | |  | **B: Elderly mice pre-immunized with DS-Cav1/Poly(I:C) boost with DS-Cav1 adjuvanted with Alum** | | | | |
| --- | --- | --- | --- | --- | --- | --- | --- | --- | --- | --- |
| Animal number | Initial study DS-Cav1+ Poly(I:C) | 85 weeks post infection | Boost 1 | Boost2 |  | Animal number | Initial study DS-Cav1+ Poly(I:C) | 85 weeks post infection | Boost 1 DS-Cav1+Alum | Boost2, DS-Cav1+Alum |
| 2726 | 6492 | 241 | 746 | 1071 |  | 9526 | 8915 | 913 | 4437 | 3820 |
| 2727 | 4624 | 1451 | 2934 | 4607 |  | 9527 | 24627 | 6368 | 8904 | 12243 |
| 2728 | 1308 | 5210 | 8283 | 8515 |  | 9528 | 21352 | 1081 | 19453 | 18440 |
| 9521 | 4937 | 7812 | 5949 | 1048 |  | 9529 | 5841 | 1279 | 2849 | 1054 |
| 9522 | 4883 | 1314 | 386 | 470 |  | 9530 | 21179 | 3532 | 10583 | 9296 |
| 9523 | 1964 | 76 | 2147 | 7235 |  | 8736 | 4799 | 10236 | 9503 | 5985 |
| 9524 | 15468 | 792 | 873 | 15338 |  | 8738 | 1458 | 56 | 3673 | 11440 |
