# Supplementary material for: Adjuvants and the vaccine response to the DS-Cav1-stabilized fusion glycoprotein of respiratory syncytial virus
Source: PLoS One. 2017 Oct 26;12(10):e0186854. doi: 10.1371/journal.pone.0186854 (PMC5658087; doi:10.1371/journal.pone.0186854)
Supplement: S5 Table — BLI analysis using DS-Cav1 site specific KO probes. (DOCX) [file pone.0186854.s005.docx]

**S5 Table. Elderly mice sera analysis using DS-Cav1 site specific KO probes: associated binding data.**

(A) DS-Cav1 adjuvanted with SAS + Carbopol Boost, elderly mice DS-Cav1 adjuvanted with SAS + Carbopol Boost, elderly mice

|  | **DS-Cav1 Detection** | | | **DS-Cav1 Site Ø KO** | | | **DS-Cav1 SiteII KO** | | | **Post** | | | **Post SiteII KO** | | |
| --- | --- | --- | --- | --- | --- | --- | --- | --- | --- | --- | --- | --- | --- | --- | --- |
| **Animal Number** | **85 Weeks p.i.** | **Boost1-SAS+Carbopol** | **Boost2-SAS+Carbopol** | **85 weeks p.i.** | **Boost1-SAS+Carbopol** | **Boost2-SAS+Carbopol** | **85 weeks p.i.** | **Boost1-SAS+Carbopol** | **Boost2-SAS+Carbopol** | **85 weeks p.i.** | **Boost1-SAS+Carbopol** | **Boost2-SAS+Carbopol** | **85 weeks p.i.** | **Boost1-SAS+Carbopol** | **Boost2-SAS+Carbopol** |
| 2726 | 0.28 | 0.99 | 1.86 | 0.27 | 0.87 | 1.58 | 0.33 | 1.09 | 1.95 | 0.10 | 0.25 | 0.62 | 0.06 | 0.16 | 0.43 |
| 2727 | 0.65 | 1.83 | 1.83 | 0.58 | 1.46 | 1.68 | 0.41 | 1.38 | 1.69 | 0.25 | 0.90 | 1.06 | 0.18 | 0.30 | 0.53 |
| 2728 | 0.72 | 2.22 | 2.39 | 0.42 | 1.90 | 2.30 | 0.68 | 2.17 | 2.58 | 0.21 | 0.83 | 1.18 | 0.19 | 0.50 | 0.71 |
| 9521 | 1.42 | 2.12 | 1.19 | 1.07 | 1.68 | 0.93 | 1.44 | 2.13 | 1.24 | 0.25 | 0.52 | 0.48 | 0.22 | 0.43 | 0.32 |
| 9522 | 0.59 | 1.76 | 1.49 | 0.31 | 1.63 | 1.47 | 0.58 | 1.92 | 1.67 | 0.19 | 0.76 | 0.64 | 0.13 | 0.69 | 0.50 |
| 9523 | 0.54 | 1.64 | 2.28 | 0.55 | 1.46 | 1.99 | 0.65 | 1.83 | 2.59 | 0.19 | 0.49 | 0.88 | 0.14 | 0.41 | 0.77 |
| 9524 | 0.69 | 0.80 | 2.62 | 0.51 | 0.47 | 2.14 | 0.64 | 0.77 | 2.79 | 0.32 | 0.19 | 0.96 | 0.23 | 0.10 | 0.85 |

(B) DS-Cav1 adjuvanted with Alum Boost, elderly mice

| **Animal Number** | **DS-Cav1 Detection** | | | **DS-Cav1 Site Ø KO** | | | **DS-Cav1 SiteII KO** | | | **Post** | | | **Post SiteII KO** | | |
| --- | --- | --- | --- | --- | --- | --- | --- | --- | --- | --- | --- | --- | --- | --- | --- |
|  | **85 Weeks p.i.** | **Boost1-Alum** | **Boost2-Alum** | **85 Weeks p.i.** | **Boost1-Alum** | **Boost2-Alum** | **85 Weeks p.i.** | **Boost1-Alum** | **Boost2-Alum** | **85 Weeks p.i.** | **Boost1-Alum** | **Boost2-Alum** | **85 Weeks p.i.** | **Boost1-Alum** | **Boost2-Alum** |
| 9526 | 1.21 | 2.00 | 2.46 | 1.05 | 1.75 | 2.22 | 0.71 | 2.09 | 2.55 | 1.03 | 0.54 | 0.71 | 0.28 | 0.33 | 0.47 |
| 9527 | 1.80 | 2.91 | 3.23 | 1.68 | 2.49 | 2.74 | 1.60 | 2.81 | 3.16 | 0.82 | 1.64 | 1.97 | 0.35 | 1.03 | 1.36 |
| 9528 | 0.60 | 1.97 | 1.64 | 0.59 | 1.34 | 1.13 | 0.62 | 1.83 | 1.64 | 0.21 | 0.78 | 0.60 | 0.19 | 0.44 | 0.35 |
| 9529 | 1.58 | 1.73 | 1.09 | 1.31 | 1.44 | 0.87 | 1.55 | 1.32 | 0.86 | 0.36 | 1.13 | 0.64 | 0.27 | 0.44 | 0.30 |
| 9530 | 1.83 | 2.77 | 2.06 | 1.73 | 2.56 | 1.90 | 1.77 | 2.68 | 1.97 | 0.77 | 1.59 | 1.19 | 0.47 | 0.83 | 0.59 |
| 8736 | 2.12 | 2.32 | 2.44 | 1.80 | 1.93 | 2.17 | 1.81 | 2.13 | 2.79 | 1.32 | 1.50 | 0.84 | 1.29 | 1.36 | 0.76 |
| 8738 | 0.28 | 2.09 | 2.28 | 0.22 | 1.78 | 1.92 | 0.30 | 2.23 | 1.96 | 0.13 | 0.60 | 1.58 | 0.13 | 0.48 | 1.32 |

BLI analysis using DS-Cav1 site specific KO probes.
