# Supplementary material for: Adjuvants and the vaccine response to the DS-Cav1-stabilized fusion glycoprotein of respiratory syncytial virus
Source: PLoS One. 2017 Oct 26;12(10):e0186854. doi: 10.1371/journal.pone.0186854 (PMC5658087; doi:10.1371/journal.pone.0186854)
Supplement: S6 Table — (DOCX) [file pone.0186854.s006.docx]

**S6 Table. ELISA measurement of IgG response in elderly mice.**

|  | **Animal number** | **Poly(I:C) 85 weeks p.i.** | | **SAS+Carbopol boost1** | | **SAS+Carbopol boost2** | |
| --- | --- | --- | --- | --- | --- | --- | --- |
| **IgG1** | 2726 | N.D. | N.D. | 256000 | 256000 | 409600 | 409600 |
|  | 2727 | 102400 | 102400 | 256000 | 102400 | 1024000 | 1024000 |
|  | 2728 | 102400 | 102400 | 102400 | 102400 | 256000 | 256000 |
|  | 9521 | 409600 | 409600 | 256000 | 256000 | 64000 | 64000 |
|  | 9522 | N.D. | N.D. | 102400 | 102400 | 409600 | 409600 |
|  | 9523 | 102400 | 102400 | 102400 | 102400 | 409600 | 409600 |
|  | 9524 | 64000 | 25600 | 64000 | 25600 | 409600 | 409600 |
| **IgG2a** | 2726 | N.D. | N.D. | 25600 | 25600 | 256000 | 256000 |
|  | 2727 | 256000 | 409600 | 64000 | 64000 | 409600 | 409600 |
|  | 2728 | 16000 | 16000 | 64000 | 64000 | 64000 | 64000 |
|  | 9521 | 102400 | 102400 | 64000 | 64000 | 64000 | 64000 |
|  | 9522 | N.D. | N.D. | 102400 | 102400 | 102400 | 102400 |
|  | 9523 | 64000 | 102400 | 64000 | 64000 | 64000 | 64000 |
|  | 9524 | 4000 | 6400 | 25600 | 25600 | 102400 | 102400 |

|  | **Animal number** | **Poly(I:C) 85 weeks p.i.** | | **Alum boost1** | | **Alum boost2** | |
| --- | --- | --- | --- | --- | --- | --- | --- |
| **IgG1** | 9526 | 409600 | 102400 | 102400 | 256000 | 409600 | 409600 |
|  | 9527 | 102400 | 256000 | 102400 | 256000 | 256000 | 102400 |
|  | 9528 | 64000 | 64000 | 102400 | 102400 | 256000 | 256000 |
|  | 9529 | 256000 | 256000 | 256000 | 256000 | 64000 | 64000 |
|  | 9530 | 25600 | 64000 | 64000 | 102400 | 102400 | 102400 |
|  | 8736 | 64000 | 25600 | 256000 | 102400 | 256000 | 256000 |
|  | 8738 | 102400 | 102400 | 64000 | 64000 | 409600 | 409600 |
| **IgG2a** | 9526 | 6400 | 6400 | 25600 | 25600 | 64000 | 64000 |
|  | 9527 | 16000 | 16000 | 25600 | 64000 | 102400 | 102400 |
|  | 9528 | 25600 | 25600 | 64000 | 64000 | 102400 | 102400 |
|  | 9529 | 25600 | 64000 | 25600 | 64000 | 64000 | 64000 |
|  | 9530 | 64000 | 64000 | 102400 | 102400 | 256000 | 256000 |
|  | 8736 | 102400 | 102400 | 64000 | 64000 | 256000 | 256000 |
|  | 8738 | 25600 | 25600 | 25600 | 25600 | 102400 | 102400 |

N.D., not determined due to insufficient serum sample availability for all of the analysis.
